# Hemoglobinuria associated with *Candidatus Mycoplasma Haemobos* in a primiparous Holstein cow in Turkey: a case report

**DOI:** 10.1007/s11259-026-11262-9

**Published:** 2026-05-16

**Authors:** Fikri Emlik, Hüsnü Furkan Şakar, Sefer Türk, Ömer Faruk Şahin, Ufuk Erol, Kürşat Altay, Alparslan Coşkun

**Affiliations:** 1 Department of Internal Medicine, Faculty of Veterinary Medicine, Sivas Cumhuriyet University, Sivas, Turkey; 2 Department of Parasitology, Faculty of Veterinary Medicine, Sivas Cumhuriyet University, Sivas, Turkey

**Keywords:** *Candidatus Mycoplasma haemobos*, Cow, Hemoglobinuria, Hemolytic anemia, Icterus

## Abstract

Bovine hemoplasmas, including *Mycoplasma wenyonii (M. wenyonii)* and *Candidatus Mycoplasma haemobos (C. M. haemobos)*, are vector-borne pathogens that can cause significant clinical diseases in cattle. This case report from Türkiye describes the clinical findings, hematological analysis, and serum biochemistry results of a cow presenting with hemoglobinuria caused by *C. M. haemobos*. Blood samples from the infected animal were analyzed using hematological, biochemical, microscopic, and molecular methods. Multiplex polymerase chain reaction assay confirmed the infection, and microscopic analysis revealed a parasitemia rate of 6.99%. Clinical signs included hemoglobinuria, icterus, and anemia. The treatment included fluid therapy, antibiotic (oxytetracycline), and antianemic drugs. The cattle died 1 month post-treatment, reportedly due to a lower respiratory tract infection. This case report describes the diagnostic methods, clinical findings, and laboratory results. A widescale molecular survey of *C. M. haemobos* in cattle is warranted, with investigation of its pathogenicity and pathogenies.

## Introduction

Hemoplasmas (hemotrophic mycoplasmas) are small, pleomorphic, and uncultivable bacteria that parasitize erythrocytes in domestic and wild animals, as well as humans (Neimark et al. 2001; Kmetiuk et al. 2025). Originally classified as *Eperythrozoon* and *Haemobartonella*, these organisms were later reassigned to the genus *Mycoplasma* according to subsequent molecular and phylogenetic analyses based on 16 S ribosomal DNA (rDNA) sequences (Su et al. 2010; Mucan and İkiz 2023; Altay et al. 2025).

Among bovine hemoplasmas, *Mycoplasma wenyonii (M. wenyonii)* and *Candidatus Mycoplasma haemobos (C. M. haemobos)* are the most commonly reported species (Díaz-Sánchez et al. 2019; Nouvel et al. 2019). These pathogens have been detected in cattle and water buffalo across regions, such as Japan, the Philippines, Mozambique, Cuba, the United Kingdom, Brazil, France, and Kyrgyzstan, primarily using polymerase chain reaction (PCR) and DNA sequencing (Tagawa et al. 2008; Ayling et al. 2012; Gonçalves et al. 2018; Díaz-Sánchez et al. 2019; de Mello et al. 2019; Nouvel et al. 2019; Ybañez et al. 2019; Altay et al. 2023).

In Türkiye, molecular detection of *M. wenyonii* and *C. M. haemobos* in cattle was first reported by Erol et al. (2023). To date, this remains the only study investigating the molecular epidemiology of bovine hemoplasma infections in the country. In that study, *Mycoplasma* spp. were detected in 31.64% (94/297) of cattle, indicating a relatively high prevalence. Hemoplasmas is thought to be transmitted by blood-feeding arthropods, including ticks, fleas, flies, and mosquitoes, and via iatrogenic routes or direct contact with infected blood (Kim et al. 2024). Transplacental transmission has also been reported (Hornok et al. 2011).

Hemoplasma infections are typically considered subclinical but can cause clinically significant disease depending on host and infection factors. Reported clinical signs in cattle include hemolytic anemia, fever, hemoglobinuria, lymphadenopathy, reduced milk production, and reproductive disorders such as infertility (Kim et al. 2024). Furthermore, hematological changes, particularly in erythrocyte indices, have been reported in infected animals.

Despite increasing molecular evidence, comprehensive reports integrating clinical, hematological, and biochemical findings of *C. M. haemobos* infection in cattle remain limited. Diagnosis of hemoplasma infections is challenging because these organisms cannot be cultured in cell-free media (Neimark et al. 2001). Although Giemsa or Romanowsky-type staining can be used, distinguishing hemoplasmas from erythrocytic inclusions, such as Pappenheimer, Heinz, and Howell–Jolly bodies, is often difficult, especially in chronic or low-parasitemia infections (Kahn 2005; Ritzmann et al. 2009). Therefore, molecular diagnostic methods, such as conventional, nested, multiplex, and real-time PCR, are widely preferred due to their higher sensitivity and specificity (Khaki et al. 2012; Ybañez et al. 2019).

## Case presentation

**Fig. 1:**
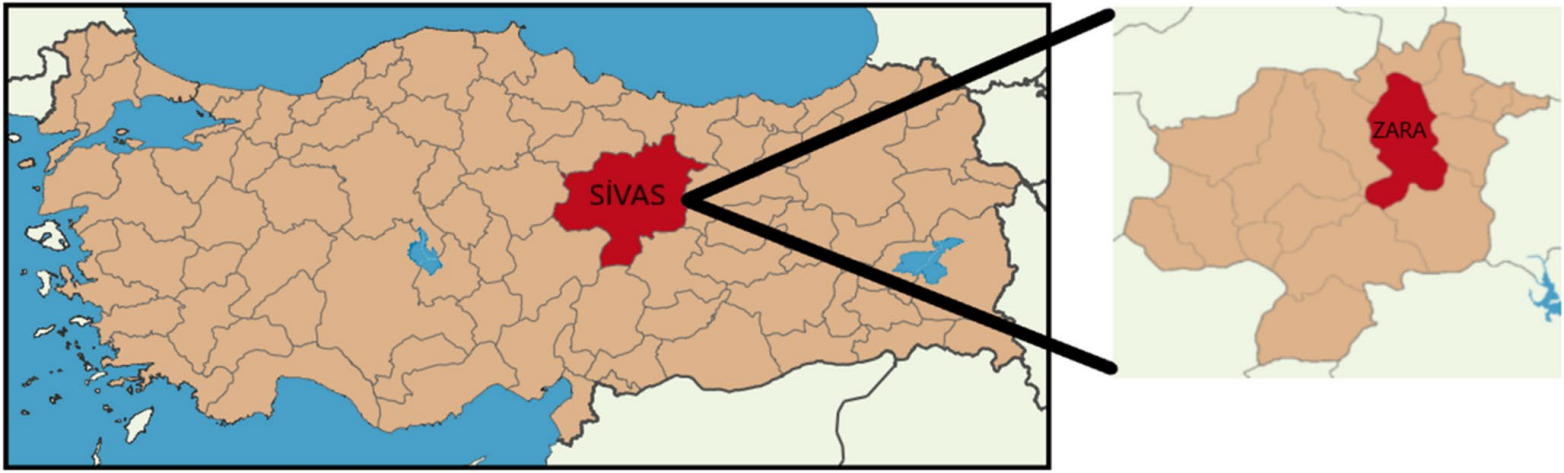
The location of Sivas province and Zara district in Türkiye

A 3-year-old primiparous Holstein cow was presented to the Internal Medicine Clinic of the Animal Hospital, Faculty of Veterinary Medicine, Sivas Cumhuriyet University, from the Zara district of Sivas, Türkiye (Fig. 1). The cow had a 1-week history of anorexia, reduced feed intake, and lethargy, hemoglobinuria over the preceding 2 days.

### Sample collection and analysis

Following physical examination, 5 mL of blood was aseptically collected from the jugular vein using an 18-G sterile needle. The sample was divided into ethylenediaminetetraacetic acid (EDTA)-containing and gel tubes. Hematological analyses were performed within 30 min using an automated veterinary hematology analyzer (Mindray BC-5000, China). The gel tube blood sample was allowed to clot at room temperature (25 °C) for 30 min and subsequently centrifuged at 3000 rpm for 10 min to obtain serum. The serum was stored at − 80 °C (Haier, China) until biochemical analysis. Biochemical parameters were analyzed using an automated analyzer (Roche Cobas 8000, Japan). After initial analyses, the remaining samples were stored at − 20 °C for genomic DNA extraction.

### Microscopic examination

Microscopic examination was performed immediately after blood collection. A thin blood smear was prepared by placing a drop of EDTA-anticoagulated blood on a clean glass slide and air-drying it at room temperature. The air-dried smear was fixed in absolute methanol for 5 min and subsequently stained with Giemsa solution (BESLAB, Ref: BS-191) for 45 min. After staining, the slide was gently rinsed with running water and air-dried. Smears were examined under a light microscope at 1000× magnification using oil immersion.

### Genomic DNA (gDNA) extraction from blood samples

Genomic DNA (gDNA) was extracted from the collected anticoagulated blood sample using a commercial kit (PureLink Genomic DNA Kit, Cat. No.: K1820-02, Invitrogen, Carlsbad, USA) according to the manufacturer’s protocol. During extraction, positive (*C. M. haemobos*, GenBank Accession Number: OM468184) and negative (DNase-RNase-free sterile water) controls were included to prevent false-positive or false-negative results. Extracted samples were stored at − 20 °C until multiplex PCR analysis.

## Molecular detection of vector-borne pathogens

Extracted DNA samples were screened for bovine hemoplasma species, *AnapLasma spp.*,* Babesia spp.*, and *Theileria spp.* using PCR assays. The primers used are listed in Table 1. PCR reactions were prepared according to the protocol described by Altay et al. (2023). A multiplex PCR assay targeting the 16 S rRNA gene was used for simultaneous detection and differentiation of bovine hemoplasma species (*M. wenyonii* and *C. M. haemobos*) using species-specific primers. In contrast, *Anaplasma spp.*,* Babesia spp.*,* and Theileria spp.* were detected using separate conventional (singleplex) PCR assays with pathogen-specific primers and optimized annealing temperatures. PCR amplification was performed in a 25 µL containing DNase- and RNase-free water (Qiagen^®^, Germany), 10× PCR buffer (Thermo Scientific™, Lithuania), MgCl₂ (25 mM; Thermo Scientific™, Lithuania), 200 µM of each deoxynucleotide triphosphate (dNTP; Thermo Scientific™, Lithuania), 1.25 U Taq DNA polymerase (Thermo Scientific™, Lithuania), 1 µL of each primer (10 pmol/µL), and 3 µL of template DNA. Thermal cycling began with initial denaturation at 94 °C for 5 min, followed by 35 cycles of denaturation at 94 °C for 1 min, annealing at 59 °C, 50 °C, and 56 °C for *Mycoplasma*,* Anaplasma*, and *Babesia–Theileria*, respectively, for 1 min, and extension at 72 °C for 1 min, with a final extension at 72 °C for 5 min. Positive controls (*M. wenyonii* GenBank accession number OM468183; *C. M. haemobos* OM468184; *Anaplasma capra* ON763216; *Babesia vogeli* OR116199) and negative controls were included in each PCR run to prevent false-positive and false-negative results. Positive control samples were obtained from archived specimens stored at − 20 °C in the Department of Parasitology, Faculty of Veterinary Medicine, Sivas Cumhuriyet University. PCR products were separated by electrophoresis on a 1.5% agarose gel at 90 V for 60 min. The gel was stained with ethidium bromide and visualized using a UV transilluminator (Altay et al. 2023).

**Table 1:** Primers used for molecular detection of vector-borne pathogens

Species	Target Gene	Primer Name	Primer Sequence	Amplicon (bp)	Tm Value (°C)	Reference
M. wenyonii	*16 S rRNA*	F2 MW-R	ACGAAAGTCTGATGGAGCAATA AGCTTYGCARTAGATTRCAAGCC	627	60 59	(Jensen et al. 2001) (Erol et al. 2023)
C. M. haemobos	*16 S rRNA*	F2 CMh-R	ACGAAAGTCTGATGGAGCAATA CTACAGCACTGAGGCTCAAAC	457	60 59	(Jensen et al. 2001) (Erol et al. 2023)
Anaplasma spp.	*16 S rRNA*	F R	AGAAGAAGTCCCGGCAAACT GAGACGACTTTTACGGATTAGCTC	800	50	(Zobba et al. 2014)
Babesia spp. Theileria spp.	*18 S rRNA*	BJ1 BN2	GTCTTGTAATTGGAATGATGG TAGTTTATGGTTAGGACTACG	500	56	(Casati et al. 2006)

## Results

### Clinical examination

**Fig. 2:**
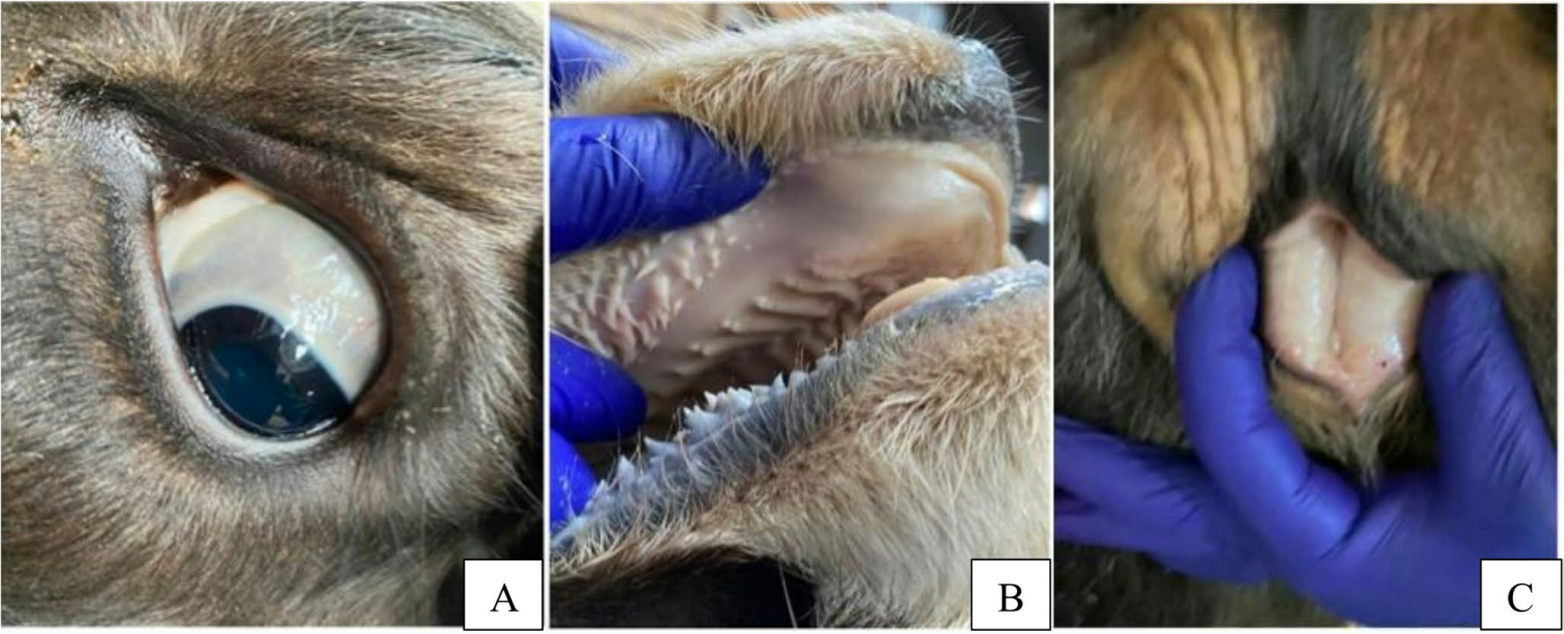
Pale and icteric appearance in the sclera (**A**), mouth (**B**) and vulva (**C**) mucosa of an infected cow with *C. M. haemobos*

Physical examination (Table 2) showed lethargy, anorexia, depression, and reduced reflexes. Hemoglobinuria was also present. Mucous membranes of the conjunctiva, oral cavity, and vulva showed pallor and icterus (Fig. 2). Additionally, dyspnea and abdominal breathing were observed.

**Table 2:** Clinical examination findings of the affected cow

Parameters	Findings
General examination	Depressed
Enophthalmos (mm)	6
Capillary refill time (s)	5
Skin recoil (s)	7
Dehydration rate (%)	10%
Rectal Temperature (°C)	39
Respiratory rate (min)	48
Heart rate (min)	124

### Hematological analysis

The hematological measurement results presented in Table 3.

**Table 3:** Hematological parameters of the affected cow

Parameters	Results	Reference Interval (Kahn 2005)
White Blood Cell Count (x10³/µL)	11.92*	5–10
Neutrophil (x10³/µL)	4.03	0.6–4.9
Lymphocyte (x10³/µL)	7.68	2.5–11.8
Monocyte (x10³/µL)	0.09	0–1.02.02
Eosynophil (x10³/µL)	0.12	0–0.35.35
Red Blod Cell Count (x10^6^/µL)	1.56**	5–10
Hemoglobin (g/dl)	3.1**	8–15
Hematocrit (%)	9.3**	24–46
MCV (fL)	60.1	40–60
MCH (pg)	19.8*	11–17
MCHC (g/dl)	32.9	31–37
RDW (%)	47.8*	17.5–26.5
Platelet Count (10³/µL)	667	100–720
MPV (fL)	5.6	4.8–7.6
PDW	15.9	12–17.5.5

Abbreviations: *MCV* mean corpuscular volume, *MCH* mean corpuscular hemoglobin, *MCHC* mean corpuscular hemoglobin concentration, *RDW* red cell distribution width, *MPV* mean platelet volume, *PDW* platelet distribution width. (* Increased value, ** Decreased value)

### Biochemical analysis

Biochemical parameters presented in Table 4.

**Table 4:** Biochemical parameters of the affected cow

Parameters	Results	Reference Interval (Kahn 2005; Khaki et al. 2012)
CREA (mg/dl)	3.36	0.50–2.20
UREA (mg/dl)	142.0*	10.0–25.0
BUN (mg/dl)	66.4*	7–25
Glucose (mg/dl)	54	40–100
Triglyceride (mg/dl)	27*	< 14
Cholesterol (mg/dl)	58**	62.0–193.0.0.0
HDL (mg/dl)	32**	51–83
LDL (mg/dl)	24	9–23
ALT (U/L)	36	7–35
AST (U/L)	258*	60–125
ALP (U/L)	57	< 500
GGT (U/L)	38*	6–17
LDH (U/L)	2179*	692–1445
CK (U/L)	957 *	< 350
T. BIL (mg/dl)	1.45*	0.01–0.5
D. BIL (mg/dl)	0.15	0.04–0.44
IND. BIL (mg/dl)	1.30*	0.03–0.06
TP (g/dl)	5.49**	6.70–7.30
ALB (g/dl)	2.19**	2.50–3.80
GLOB (g/dl)	3.30	3.0–3.5.0.5
CRP (mg/l)	0.03	0–5
Na (mmol/l)	139	136–144
Cl (mmol/l)	89**	99–107
K (mmol/l)	4.27	3.6–4.9
P (mg/dl)	7.52	5.60–8.00.60.00
Ca (mg/dl)	9.56	8.00–11.40.00.40
Mg (mg/dl)	2.03	1.50–2.90

Abbreviations: *Crea* creatinine, *Urea *urea, *BUN* blood urea nitrogen, *Glu* glucose, *TG* triglycerides, *TChol* total cholesterol, *HDL* high-density lipoprotein, *LDL* low-density lipoprotein, *ALT* alanine aminotransferase, *AST* aspartate aminotransferase, *ALP* alkaline phosphatase, *GGT* gamma-glutamyl transferase, *LDH* lactate dehydrogenase, *CK* creatine kinase, *TBil* total bilirubin, *DBil* direct bilirubin, *IBil* indirect bilirubin, *TP* total protein, *Alb* albumin, *Glob* globulin, *CRP* C-reactive protein, *Na* sodium, *Cl* chloride, *K* potassium, *P* phosphorus, *Ca* calcium, *Mg* magnesium. (* Increased value, ** Decreased value)

### Microscopic analysis

**Fig. 3:**
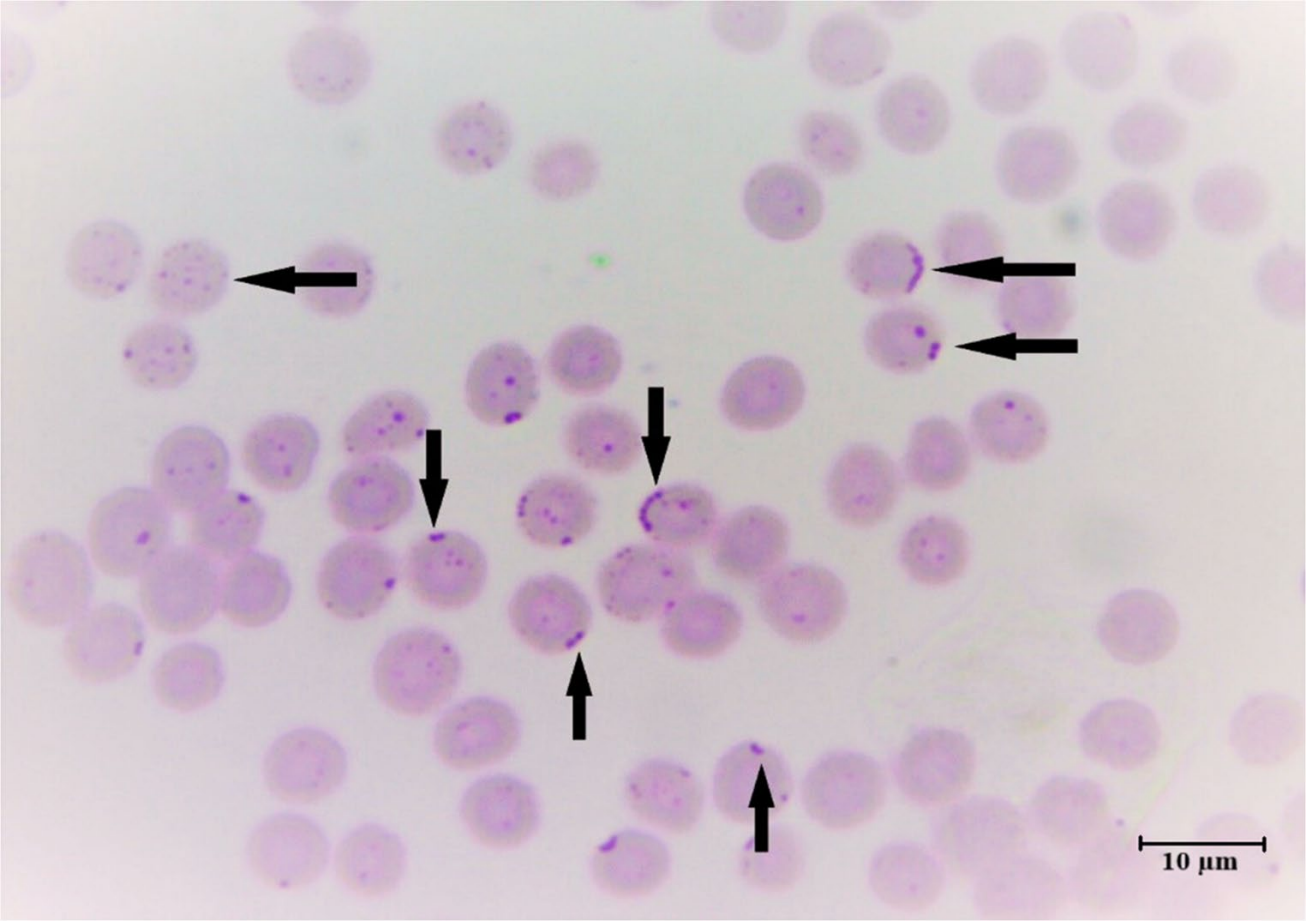
The microscopic appearance of the blood film shows a high load of*Mycoplasma*-like structures present on red blood cells 1000× (Giemsa staining)

On Giemsa-strained blood smears examined under a light microscope at 1000× magnification, spherical hemoplasmas were observed on erythrocyte surfaces, either individually or in chains (Fig. 3). No other vector-borne blood pathogens, such as *Babesia* spp., *Theileria* spp., or *Anaplasma* spp., were detected. Parasitemia was determined by examining 50 microscopic fields and counting the total erythrocytes and infected erythrocytes. Consequently, based on microscopic examination, the parasitemia rate was calculated as 6.99%.

### Molecular analysis

**Fig. 4:**
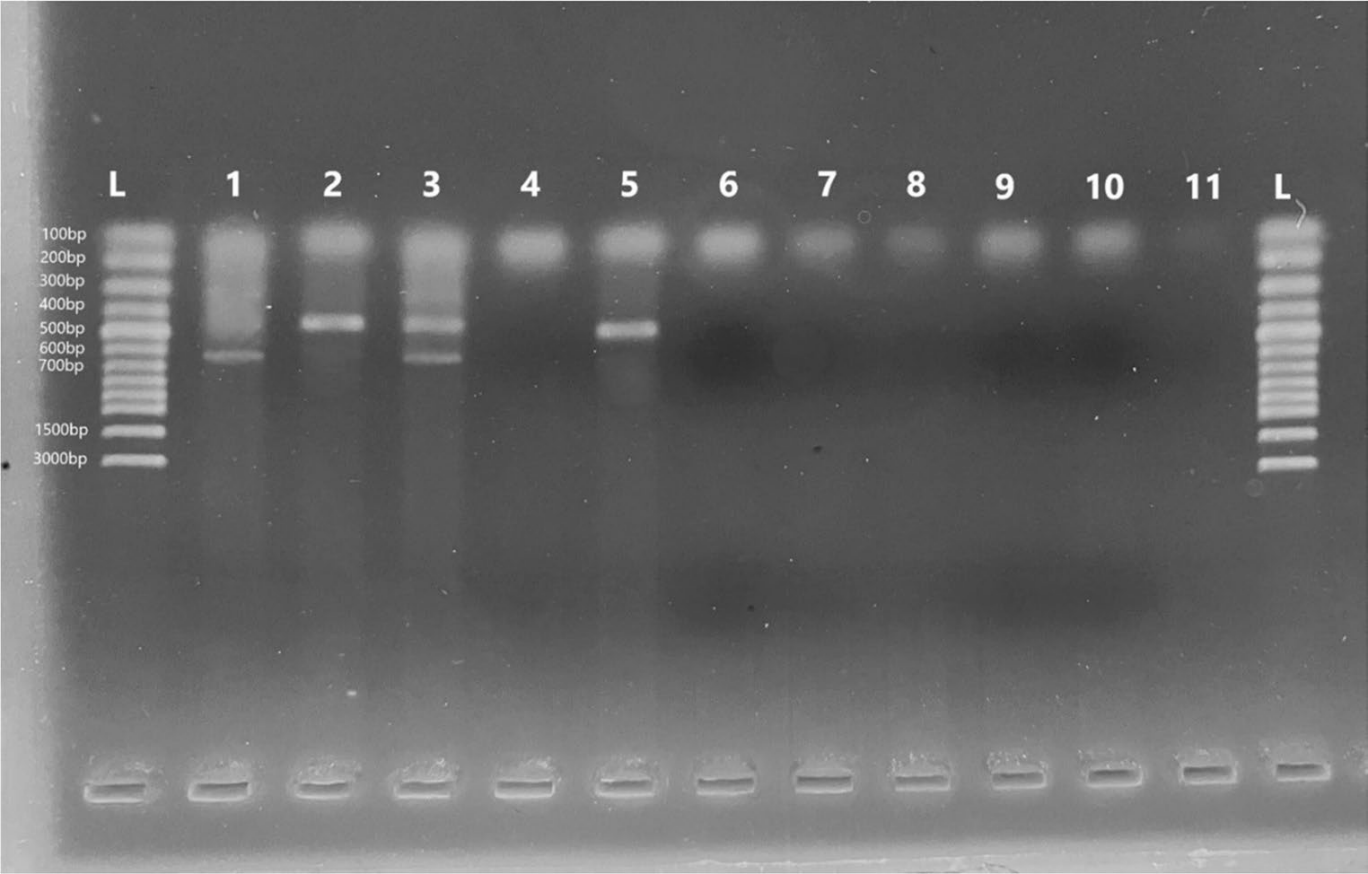
PCR products of a tested cow with haemoplasma species. L. Ladder, 1. *M. wenyonii *positive control, 2. *C. M. haemobos* positive control, 3. Positive control of mix haemoplasma species, 4. Negative control, 5. *C. M. haemobos* positive cow sample.

gDNA was extracted from blood samples and screened for bovine hemoplasma species, *Anaplasma* spp., *Babesia* spp., and *Theileria*spp.using PCR assay. The cattle with clinical signs was identified as having *C.M. haemobos* based on the multiplex PCR results (Figure 4). No other pathogens were detected in the cow.

### Treatment

Treatment was initiated with intravenous fluid therapy (Polifleks Izolen Balanced Electrolyte Solution, Polifarma; %5 Dextrose, Polifarma; Isotonic Saline, Polifarma) to relieve dehydration. To manage*Mycoplasma* infection, long-acting oxytetracycline (Oxtra LA, Fatro) was administered intramuscularly at 20 mg/kg. A second dose at the same concentration was administered 3 days after the initial treatment. Additionally, antianemic therapy, including iron and vitamin B12 combination (Fercobsang and Vetoquinol), was administered to manage anemia. Following treatment, the cattle returned to a normal clinical condition within 20 days, based on physical examination findings. However, recovery was not scientifically confirmed by follow-up laboratory testing, as the owner declined repeat hematological and biochemical analyses due to clinical improvement (resumption of appetite, rumination, and weight gain), a limitation of this case. Furthermore, the cow died 1 month post-treatment trial, reportedly due to a lower respiratory tract infection according to the owner.

## Discussion

*Hemoplasma spp.* infections caused by *M. wenyonii* and *C. M. haemobos* have been reported in various hosts, including cattle, buffalo, and deer, across multiple regions worldwide (Ade et al. 2018; Díaz-Sánchez et al. 2019; Galon et al. 2020; Boularias et al. 2020). However, data on bovine hemoplasma infections in Türkiye remain limited. Previous studies have reported *M. wenyonii* using microscopic examination (Şaki and Özer, 2009) and PCR-based detection in small ruminants (Aktaş and Özübek, 2017). More recently, molecular studies have confirmed the presence and prevalence of both *M. wenyonii* and *C. M. haemobos* in cattle and water buffalo in Türkiye (Erol et al. 2023). Therefore, identifying the pathogen and assessing its clinical relevance are important Although hemoplasma infections are typically subclinical, overt clinical disease is strongly influenced by host-related factors. Young age, physiological stress, concurrent diseases, dehydration, immunosuppression, and high parasitemia are key factors associated with disease manifestation (Hoelzle et al. 2011; Merck Veterinary Manual, 2024). In this case, marked hemolytic anemia, severe dehydration, metabolic disturbances, and a relatively high parasitemia rate (6.99%) likely contributed to progression from a typically subclinical infection to a clinically evident condition. These findings suggest that disease severity depends not only on pathogen presence but also on host physiological status. 

Previous studies on *C. M. haemobos* vary substantially in design and clinical context. Molecular surveys in apparently healthy cattle have mainly focused on prevalence, providing limited data on clinical findings or parasitemia levels (Niethammer et al. 2018; Díaz-Sánchez et al. 2019; Kim et al. 2024). In contrast, case reports of clinically affected animals have described anemia and hemoglobinuria; however, quantitative parasitemia data are often not been reported (Hofmann-Lehmann et al. 2004; Hoelzle et al. 2011; Tagawa et al. 2012; McFadden et al. 2016). This case contributes to the literature by combining clinical findings with detailed hematological, biochemical, and quantitative parasitemia data, suggesting that parasite burden may influence clinical severity. 

The relationship between hemoplasma infection and anemia remains controversial. While some studies report no significant association (Niethammer et al. 2018; Díaz-Sánchez et al. 2019; Kim et al. 2024), others have observed hematological alterations in infected animals. In this, red blood cell (RBC) and hematocrit values were markedly lower (1.56 × 10⁶/µL and 9.3%, respectively) compared to previously reported values, which may be attributed to the higher parasitemia level. 

Additionally, slight changes in MCV and MCH values were observed, suggesting that hemoplasma infections may affect erythrocyte indices. Differences between studies may reflect variation in anemia type (regenerative vs. non-regenerative), host response, and infection severity. 

Biochemical findings were most consistent with prerenal azotemia, with elevated serum urea and creatinine levels, indicating decreased renal perfusion, particularly due to dehydration and hypovolemia (Levey et al. 1988; Rose, 2011). The marked dehydration, prolonged capillary refill time, and enophthalmos strongly support a prerenal origin. In addition, intravascular hemolysis may have increased nitrogenous waste products and interfered with biochemical interpretation, further complicating assessment (De Scally et al. 2004; Defauw et al. 2018). The concurrent hypochloremia likely reflects reduced intake and fluid shifts rather than intrinsic renal dysfunction. 

Severe intravascular hemolysis involves release of free hemoglobin into circulation; when renal reabsorptive capacity is exceeded, hemoglobinuria occurs (Rother et al. 2005). In this case, hemoglobinuria, hyperbilirubinemia, and icterus indicate ongoing hemolysis, while increased lactate dehydrogenase (LDH) levels likely indicate erythrocyte destruction, due to its high concentration in RBCs (Turgut 2000). Although creatine kinase (CK) is a marker of muscle damage, hemolysis can cause analytical interference, resulting in falsely elevated values. In the absence of myopathy, the increased LDH and CK levels in this case are more likely secondary to hemolysis rather than primary muscle injury. These findings underscore the importance of interpreting biochemical results in clinical context.

This case has several limitations. As a single case report, its findings may not be generalizable. The lack of long-term follow-up limits assessment of disease progression and prognosis, while the absence of comparative data from healthy or other affected individuals restricts the interpretation of observed alterations. Urine analysis was not performed, limiting a more comprehensive evaluation of renal function. Finally, the lack of herd-level investigation precluded assessment of potential environmental or epidemiological factors

## Conclusion

In conclusion, this case report presents, for the first time in Türkiye, the clinical, hematological, and biochemical findings associated with*C. M. haemobos* infection in cattle. Although hemoplasma infections are often subclinical, high parasitemia levels may be associated with severe clinical disease, including hemolytic anemia, icterus, and hemoglobinuria. Therefore, *C.M. haemobos* should be included in the differential diagnosis of cattle presenting with these clinical signs.

## Data Availability

No datasets were generated or analysed during the current study.

## References

[CR1] Ade J, Niethammer F, Schade B et al (2018) Quantitative analysis of *Mycoplasma wenyonii* and ‘*Candidatus Mycoplasma haemobos*” infections in cattle using novel gapN-based realtime PCR assays. Vet Microbiol 220:1–6. 10.1016/j.vetmic.2018.04.028 29885793 10.1016/j.vetmic.2018.04.028

[CR37] Șakİ CE, Özer E (2009) Clinical Eperythrozoon wenyoni (Adler and Ellenbogen, 1934) and Haemobartonella bovis (Donatin and Lestoquard, 1934) infection in a cow

[CR2] Aktas M, Ozubek S (2017) A molecular survey of small ruminant hemotrophic mycoplasmosis in Turkey, including first laboratory confirmed clinical cases caused by *Mycoplasma ovis*. Vet Microbiol 208:217–222. 10.1016/j.vetmic.2017.08.011 28888641 10.1016/j.vetmic.2017.08.011

[CR3] Altay K, Coskun A, Erol U et al (2025) Development of a novel triplex-PCR assay for the identification of feline hemoplasma species and survey of hemoplasma species in cats in Türkiye. Parasitol Int 104:102969. 10.1016/j.parint.2024.102969 39276922 10.1016/j.parint.2024.102969

[CR4] Altay K, Sahin OF, Erol U, Aytmirzakizi A (2023) First molecular detection and phylogenetic analysis of *Mycoplasma wenyonii* and *Candidatus Mycoplasma haemobos* in cow in different parts of Kyrgyzstan. Biol (Bratisl) 78:633–640. 10.1007/s11756-022-01292-4

[CR5] Ayling RD, Bisgaard-Frantzen S, Adler A et al (2012) Detection of’Candidatus Mycoplasma haemobos’, Mycoplasma wenyonii and Anaplasma phagocytophilum from cow in England. Vet Record 170:543a. 10.1136/vr.100636 10.1136/vr.100636 22517868

[CR6] Boularias G, Azzag N, Gandoin C et al (2020) Bovines harbor a diverse array of vector-borne pathogens in Northeast Algeria. Pathogens 9:883. 10.3390/pathogens9110883 33113771 10.3390/pathogens9110883 PMC7692033

[CR7] Casati S, Sager H, Gern L, Piffaretti J-C (2006) Presence of potentially pathogenic *Babesia* sp. for human in *Ixodes ricinus* in Switzerland. Ann Agric Environ Med 13:65–70 16841874

[CR11] Díaz-Sánchez AA, Corona-González B, Meli ML et al (2019) First molecular evidence of bovine haemoplasma species (Mycoplasma spp.) in water buffalo and dairy cow herds in Cuba. Parasit Vectors 12:78. 10.1186/s13071-019-3325-y 30732656 10.1186/s13071-019-3325-y PMC6367761

[CR10] Defauw P, Daminet S, Leisewitz AL et al (2018) Renal azotemia and associated clinical and laboratory findings in dogs with Babesia rossi infection. Vet Parasitol 260:22–29. 10.1016/j.vetpar.2018.07.012 30197009 10.1016/j.vetpar.2018.07.012

[CR8] de Mello VVC, de Souza Ramos IA, Herrera HM et al (2019) Occurrence and genetic diversity of haemoplasmas in beef cow from the Brazilian Pantanal, an endemic area for bovine trypanosomiasis in South America. Comp Immunol Microbiol Infect Dis 66:101337. 10.1016/j.cimid.2019.101337 31437678 10.1016/j.cimid.2019.101337

[CR9] De Scally MP, Lobetti RG, Reyers F, Humphris D (2004) Are urea and creatinine values reliable indicators of azotaemia in canine babesiosis? J S Afr Vet Assoc 75:121–124. https://hdl.handle.net/10520/EJC99576 15628803 10.4102/jsava.v75i3.466

[CR12] Erol U, Sahin OF, Altay K (2023) Molecular prevalence of bovine hemoplasmosis in Turkey with first detection of Mycoplasma wenyonii and Candidatus Mycoplasma haemobos in cow and water buffalo. Vet Res Commun 47:207–215. 10.1007/s11259-022-09943-2 35624402 10.1007/s11259-022-09943-2

[CR14] Galon EMS, Ybañez RHD, Moumouni PFA et al (2020) Molecular survey of tick-borne pathogens infecting backyard cow and water buffaloes in Quezon province, Philippines. J Vet Med Sci 82:886–890. 10.1292/jvms.19-0636 32418944 10.1292/jvms.19-0636 PMC7399310

[CR16] Gonçalves LR, Teixeira MMG, Rodrigues AC et al (2018) Molecular detection of Bartonella species and haemoplasmas in wild African buffalo (Syncerus caffer) in Mozambique, Africa. Parasitol Open 4:1–8. 10.1017/pao.2018.10

[CR17] Hoelzle K, Winkler M, Kramer MM et al (2011) Detection of Candidatus Mycoplasma haemobos in cow with anaemia. Vet J 187:408–410. 10.1016/j.tvjl.2010.01.016 20188610 10.1016/j.tvjl.2010.01.016

[CR18] Hofmann-Lehmann R, Meli ML, Dreher UM et al (2004) Concurrent infections with vector-borne pathogens associated with fatal hemolytic anemia in a cow herd in Switzerland. J Clin Microbiol 42:3775–3780. 10.1128/jcm.42.8.3775-3780.2004 15297529 10.1128/JCM.42.8.3775-3780.2004 PMC497630

[CR19] Hornok S, Micsutka A, Meli ML et al (2011) Molecular investigation of transplacental and vector-borne transmission of bovine haemoplasmas. Vet Microbiol 152:411–414. 10.1016/j.vetmic.2011.04.031 21605950 10.1016/j.vetmic.2011.04.031

[CR20] Jensen WA, Lappin MR, Kamkar S, Reagan WJ (2001) Use of a polymerase chain reaction assay to detect and differentiate two strains of *Haemobartonella felis* in naturally infected cats. Am J Vet Res 62:604–608. 10.2460/ajvr.2001.62.604 11327472 10.2460/ajvr.2001.62.604

[CR21] Kahn CM (2005) The Merck Veterinary Manual, 9th ed. Merck & CO. INC, White house station, NJ, USA, pp 2201–2206

[CR22] Khaki Z, Khazraiinia P, Chegini S, Khazraee Nia S (2012) Comparative study of serum lipid profile in chicken, ostrich, cow, and sheep. Comp Clin Pathol 21:259–263. 10.1007/s00580-010-1088-0

[CR23] Kim Y, Kim H, Choi J-H et al (2024) Preliminary report of *Mycoplasma wenoynii* and *Candidatus Mycoplasma haemobos* infection in Korean native cattle. BMC Vet Res 20:121. 10.1186/s12917-024-03976-2 38532391 10.1186/s12917-024-03976-2 PMC10964582

[CR24] Kmetiuk LB et al (2025) Hemotropic mycoplasmas (hemoplasmas) in indigenous populations and their dogs living in reservation areas, Brazil. Sci Rep 15:7973. 10.1038/s41598-025-87938-0 40055404 10.1038/s41598-025-87938-0 PMC11889244

[CR25] Levey AS, Perrone RD, Madias NE (1988) Serum creatinine and renal function. Annu Rev Med 39:465–490. 10.1146/annurev.me.39.020188.002341 3285786 10.1146/annurev.me.39.020188.002341

[CR26] McFadden AMJ, Ha HJ, Donald JJ et al (2016) Investigation of bovine haemoplasmas and their association with anaemia in New Zealand cow. N Z Vet J 64:65–68. 10.1080/00480169.2015.1090356 26411673 10.1080/00480169.2015.1090356

[CR27] Merck Veterinary Manual (2024) Hemotropic Mycoplasma infections in animals. MSD Veterinary Manual. https://www.msdvetmanual.com/circulatory-system/blood-parasites/hemotropic-mycoplasma-infections-in-animals

[CR28] Mucan T, İkiz S (2023) Sığırlarda Mycoplasma wenyonii ve Candidatus Mycoplasma Haemobos. Istanbul Rumeli Univ Saglik Bilim Derg 2:66–77

[CR30] Neimark H, Johansson K-E, Rikihisa Y, Tully JG (2001) Proposal to transfer some members of the genera *Haemobartonella* and *Eperythrozoon* to the genus *Mycoplasma* with descriptions of ‘Candidatus *Mycoplasma haemofelis*’, ‘Candidatus *Mycoplasma haemomuris*’, ‘Candidatus *Mycoplasma haemosuis*’ and ‘Candidatus *Mycoplasma wenyonii*.’ Int J Syst Evol Microbiol 51:891–899. 10.1099/00207713-51-3-891 11411711 10.1099/00207713-51-3-891

[CR31] Niethammer FM, Ade J, Hoelzle LE, Schade B (2018) Hemotrophic mycoplasma in Simmental cattle in Bavaria: prevalence, blood parameters, and transplacental transmission of ‘Candidatus *Mycoplasma haemobos*’ and *Mycoplasma wenyonii*. Acta Vet Scand 60:74. 10.1186/s13028-018-0428-y 30445976 10.1186/s13028-018-0428-y PMC6240245

[CR32] Nouvel LX, Hygonenq M-C, Catays G et al (2019) First detection of *Mycoplasma wenyonii* in France: identification, evaluation of the clinical impact and development of a new specific detection assay. Comp Immunol Microbiol Infect Dis 63:148–153. 10.1016/j.cimid.2019.01.010 30961812 10.1016/j.cimid.2019.01.010

[CR34] Ritzmann M, Grimm J, Heinritzi K et al (2009) Prevalence of *Mycoplasma suis* in slaughter pigs, with correlation of PCR results to hematological findings. Vet Microbiol 133:84–91. 10.1016/j.vetmic.2008.06.015 18687536 10.1016/j.vetmic.2008.06.015

[CR244444] Rother RP, Bell L, Hillmen P, Gladwin MT (2005) The clinical sequelae of intravascular hemolysis and extracellular plasma hemoglobin: a novel mechanism of human disease. Jama, 293(13), 1653–1662. 10.1001/jama.293.13.1653 15811985

[CR38] Su QL, Song HQ, Lin RQ et al (2010) The detection of Candidatus Mycoplasma haemobos in cow and buffalo in China. Trop Anim Health Prod 42:1805–1808. 10.1007/s11250-010-9640-0 20596775 10.1007/s11250-010-9640-0

[CR39] Tagawa M, Matsumoto K, Inokuma H (2008) Molecular detection of *Mycoplasma wenyonii* and ‘*Candidatus Mycoplasma haemobos*’ in cow in Hokkaido, Japan. Vet Microbiol 132:177–180. 10.1016/j.vetmic.2008.05.006 18571343 10.1016/j.vetmic.2008.05.006

[CR40] Tagawa M, Ybanez AP, Matsumoto K et al (2012) Prevalence and risk factor analysis of bovine haemoplasma infection by direct PCR in Eastern Hokkaido, Japan. J Vet Med Sci 74:1171–1176. 10.1292/jvms.12-0118 22673725 10.1292/jvms.12-0118

[CR41] Turgut K (2000) Veteriner Klinik Laboratuvar Teşhis, 2nd Ed. Press Bahçıvanlar A.Ş., Konya

[CR42] Ybañez AP, Ybañez RHD, Armonia RKM et al (2019) First molecular detection of *Mycoplasma wenyonii* and the ectoparasite biodiversity in dairy water buffalo and cattle in Bohol, Philippines. Parasitol Int 70:77–81. 10.1016/j.parint.2019.02.004 30776450 10.1016/j.parint.2019.02.004

[CR43] Zobba R, Anfossi AG, Pinna Parpaglia ML et al (2014) Molecular investigation and phylogeny of *Anaplasma* spp. in Mediterranean ruminants reveal the presence of neutrophil-tropic strains closely related to *A. platys*. Appl Environ Microbiol 80:271–280. 10.1128/AEM.03129-13 24162569 10.1128/AEM.03129-13 PMC3911010

